# Edge state magnetism in zigzag-interfaced graphene via spin susceptibility measurements

**DOI:** 10.1038/srep13382

**Published:** 2015-08-26

**Authors:** T. L. Makarova, A. L. Shelankov, A. A. Zyrianova, A. I. Veinger, T. V. Tisnek, E. Lähderanta, A. I. Shames, A. V. Okotrub, L. G. Bulusheva, G. N. Chekhova, D. V. Pinakov, I. P. Asanov, Ž. Šljivančanin

**Affiliations:** 1 Lappeenranta University of Technology, FI-53851 Lappeenranta, Finland; 2 Ioffe Physical Technical Institute, Polytechnicheskaya 26, 194021 St. Petersburg, Russia; 3 Ben-Gurion University of the Negev, 84105, Beer-Sheva, Israel; 4 Nikolaev Institute of Inorganic Chemistry SB RAS, 630060, Novosibirsk, Russia; 5 Vinča Institute of Nuclear Sciences, P.O. Box 522, RS-11001 Belgrade, Serbia

## Abstract

Development of graphene spintronic devices relies on transforming it into a material with a spin order. Attempts to make graphene magnetic by introducing zigzag edge states have failed due to energetically unstable structure of torn zigzag edges. Here, we report on the formation of nanoridges, i.e., stable crystallographically oriented fluorine monoatomic chains, and provide experimental evidence for strongly coupled magnetic states at the graphene-fluorographene interfaces. From the first principle calculations, the spins at the localized edge states are ferromagnetically ordered within each of the zigzag interface whereas the spin interaction across a nanoridge is antiferromagnetic. Magnetic susceptibility data agree with this physical picture and exhibit behaviour typical of quantum spin-ladder system with ferromagnetic legs and antiferromagnetic rungs. The exchange coupling constant along the rungs is measured to be 450 K. The coupling is strong enough to consider graphene with fluorine nanoridges as a candidate for a room temperature spintronics material.

The search for evidence of the magnetic ordering in graphene has been an active field of research for many years and has focused primarily on point and line defects as the most promising candidates for engineering magnetism in graphene^1^. Recent experiments have indicated that point defects, acting as paramagnetic centres are unlikely to lead to magnetic ordering because their density appears to be limited to one Bohr magneton per approximately 1000 carbon atoms^2^. Theoretical predictions of one-dimensional (1D) ferromagnetism in graphene nanoribbons^3^,^4^ have triggered an extensive search for magnetism in graphene nanostructures with reduced dimensionality. Despite the remarkable progress achieved in the detection^5^,^6^,^7^ of localized electronic states at zigzag edges of graphene^8^, a wide and discouraging gap still exists between the theoretical anticipation of edge spin ferromagnetism and its experimental realisation. Magnetic order on the zigzag graphene edges was deduced from the transport and scanning tunnelling microscopy data^6^,^9^, but since all these measurements were done using charge-based instead of spin-based techniques, they could be a subject to various interpretations. Fortunately, a torn edge is not a prerequisite for such edge magnetism: the edges of graphene nanoribbons (GNRs) embedded in a functionalized C-sp^3^ layer possess essentially the same electronic properties as those of a freestanding GNR. Theory suggests that spin ordering can be induced along the interfaces^10^ and within relatively small 1-D and 2-D graphene nanosegments in the interior of functionalized graphene^11^. Here, we show experimentally that a dense network of fluorine chains effectively transforms an extended honeycomb lattice of sp^2^-carbon atoms into nanosegments of pristine graphene framed by C-sp^2^/C-sp^3^ interfaces. In these confined geometries, with a large interface-to-area ratio, we observe magnetic effects consistent with theories that treat edge-inherited magnetism as an essentially 1D quantum phenomenon and predict a spin-gapped ground state with an antiferromagnetic inter-edge superexchange interaction^1^,^12^,^13^,^14^,^15^,^16^. The observed magnetism is preserved up to the fluorine detachment temperature (520 K), at which the samples irreversibly collapse into a diamagnetic state.

The functionalization of graphene with fluorine transforms carbon atoms from C-sp^2^ to CF-sp^3^ hybridized states. By virtue of its favourable characteristics, the room-temperature fluorination of intercalated graphite has emerged as an efficient method of creating crystallographically selective patterns at decoupled carbon planes^17^,^18^. First, because of the enlarged interlayer spacing, both sides of the graphene layers are available to the fluorinating agent. Second, over an extended period (on the scale of several months) at room temperature, the fluorine adatoms relax into an energetically preferable configuration on the basal planes. Fluorine adsorbates tend to align in a chain sequence in which F adatoms are located on alternating sides of a graphene plane^11^,^19^. Simultaneous F adsorption at multiple sites results in the seeding of the chains in a random distribution across the graphene planes. A nuclear magnetic resonance study has indicated homogeneous fluorination and a planar configuration of the graphene sheets^18^, and angle-resolved X-ray absorption spectroscopy has provided evidence of the sequential attachment of fluorine atoms to opposite sides of a sheet^17^. Adjacent CF chains are well separated, thus creating a low-dimensional π-electron system between them.

We investigated samples with C_4_F, C_3_F and C_2_F stoichiometries; the various types of samples are detailed in Table S1 of the Supplementary Material together with their exact compositions and the intercalating molecules (guests). C_n_F compounds represent stacks of fluorinated graphene bilayers separated by guests, which are weakly diamagnetic and otherwise magnetically inert (Fig. 1a). Experimental data were collected from more than 50 samples that differ in both fluorine content and the properties of the guest molecules: small and large, polar and non-polar, organic and inorganic. Measurements of the magnetic susceptibilities demonstrated that the chemical composition of the guests does not affect the magnetism. Here, we restrict ourselves to bilayer graphenes prepared from the 2^nd^ stage graphite intercalated compounds with dichloromethane, dichloroethane, acetonitrile, acetone, and n-hexane. We have similar magnetic results on single-layer graphenes prepared from the 1^st^ stage graphite intercalated compounds, which will be reported elsewhere. The presence of guest molecules increases the distance between planes by up to ~1 nm, excluding any significant hybridisation between electronic states at different planes. Thus, the guests convert graphite to graphenes and effectively reduce the dimensionality of the electronic system from three to two. The fluorine chain patterns further reduce the effective dimensionality of the electron gas to one. Atomic force microscopy (Fig. 1b) revealed bright and dark strips that correspond to the fluorine chains and pristine graphene regions, respectively (Fig. 1c). The observed relative orientation of the strips implies that both zigzag and armchair fluorine chains are present. Using a set of spectroscopic data^17^,^18^, we determined that the chains are monatomic, and the chain length typically comprise 6–8 fluorine atoms. These crystallographically oriented fluorine chains divide the basal plane into a dense, non-periodic array of π-electron nanofragments: narrow GNR and nanosegments in the case of a low-fluorination matrix (Fig. 1d,e) and carbon polyacetylene-like nanochains when the stoichiometry approaches C_2_F (Fig. 1f).

Magnetic measurements revealed strong magnetism in these purely organic graphene-based structures. We begin by presenting results for the samples with lower fluorine contents, i.e., C_4_F and C_3_F (Fig. 2a,b). These samples exhibit a reversible magnetic response and a linear magnetic susceptibility


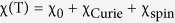


that can be split into a temperature-independent term χ_0_, a small Curie-like component χ_Curie_ (identified by the low-T upturn), and a strong non-Curie paramagnetic spin susceptibility χ_spin_. When the first two terms of Eq. 1 are subtracted, a broad maximum at 250 K followed by a low-temperature exponential decrease is revealed in χ_spin_(T), reflecting the low-dimensional nature of the spin system. The major characteristics of the thermomagnetic curves are reminiscent of molecule-based magnets^20^, particularly organic radical crystals^21^ in which interactions are constrained to act in fewer than three spatial dimensions.

The behaviour of the C_3_F samples is similar to the C_4_F samples. The results obtained for the C_2_F samples in which the C-sp^2^-islands shrink into the C-sp^2^-chains, highlight the critical role played by the fluorine patterns in the observed magnetism (Fig. 2c). An exponential trend in susceptibility is observed below a kink at T ≈ 150 K; near the kink, thermal hysteresis is observed, with a temperature difference of T ≈ 20 K between the heating and cooling traces. Curie paramagnetism is undetectable suggesting the near absence of isolated spins in the dense CF network, whereas the magnetic response contains a small irreversible contribution (Fig. 2c, inset) which depends weakly on temperature.

Figure 2d presents a comparison of the normalized spin-susceptibility curves for the three stoichiometries. The logarithmic plots of χ_spin_ *vs* 1/T (Fig. 2d, inset) clearly demonstrate exponentially activated susceptibility (χ_spin_ ~ exp (−Δ/T)) and thus reveal an energy gap Δ — the so-called spin gap — in the spin excitation spectrum. The existence of the spin gap is an unequivocal manifestation of spin-spin interaction. However, long-range antiferromagnetic spin ordering is very unlikely in our samples: the absence of sharp features in χ(T) suggests that, if we are encountering a cooperative phenomenon, only a few atoms are correlated. The spin gap and the absence of classical spin ordering are purely quantum phenomena, which are relatively common at low dimensions, where quantum and/or thermal fluctuations suppress magnetic order. The simplest model of an activated magnetic susceptibility is the scenario in which the spins are antiferromagnetically coupled pairwise as dimers^22^. For our description of the magnetism in fluorinated graphene, we will rely on the more sophisticated spin-ladder model^23^ for which spin dimers and linear chains are the limiting cases.

Fluorination is well known to be an effective method of introducing localized spin centres into graphene^2^. Obviously, the magnetism observed in our experiments originates from structures more complex than monatomic adsorbates, and we will demonstrate that the fluorine chains provide conditions that favour spontaneous spin alignment. The model of the magnetism, as described below and in the Supplementary Material, is based on the fact that carbon atoms that are covalently bonded to fluorines lack π-electrons; i.e., the fluorine chain is seen by the graphene π-electron network as a high-energy barrier — a “nanoridge” extending the length of the chain. Thus, the fluorine chain serves as chemical scissors that cut the graphene plane in the sense that the lattice sites that are occupied by CF groups become unavailable to the π-electron system. This “cut” creates two edges that are separated by a nearly impenetrable CF-nanoridge. Graphene bipartite lattice consists of inequivalent α and β sublattices. In the case of a zigzag chain, one expects a set of localized spin states in sublattice α on one side of the nanoridge and sublattice β on the other side.

In the simplest model, we adopt a picture of N_s_ spins ½ localized on each side of the ridge, where N_s_ is of the order of the number of fluorines in the chain. Because of the ferromagnetic intra-edge exchange interaction^1^, the two sets of edge spins behave as collective spins, |**S**_1,2_| = ½N_s_. The small but finite penetrability of the nanoridge allows a superexchange antiferromagnetic interaction across the edges, which favours an antiparallel orientation of the edge spins, **S**_1_ = **−S**_2_, or, more precisely, a gapped singlet ground state. The existence of the spin gap in the energy spectrum of the nanoridge is a common feature of a system with a spontaneously broken symmetry at infinite volume, where the broken symmetry is restored by quantum fluctuations at finite size^24^,^25^.

This interaction resembles the exchange couplings between the zigzag edge-inherited states in GNRs, which were described using a spin-ladder model shortly after their theoretical discovery^12^. The difference between the nanoridge model and the GNR model lies in the microscopic origins of the interactions: the antiferromagnetic-like interaction originates not from the bulk tails of the edge states^26^ but from the overlapping of the edge states on the nanoridge separating the two edges.

To verify this qualitative picture of the magnetism in fluorinated graphene, we performed density functional theory (DFT) calculations using GPAW computer code^27^. First, we applied DFT to illuminate the origin of the magnetism in graphene functionalized with fluorine, guided by the experimental evidence that monatomic chains of fewer than 10 F atoms on graphene, are dominant structural motifs^17^,^18^. The DFT calculations for well-separated F lines and chains of four to ten atoms clearly revealed the emergence of magnetism in fluorinated graphene, as demonstrated in Fig. 3a, where we plot the spin density in the vicinity of a zigzag chain with eight F atoms. The calculated complex magnetic configuration combines the strong ferromagnetic interaction between the local magnetic moments of C atoms on the same side of the chain with the antiferromagnetic coupling between the magnetic moments of C atoms located on different sides of the CF chain. According to our calculations, no similar magnetic effects occur in isolated armchair chains of F atoms. After we confirmed that distant zigzag F chains induce magnetism in graphene, we investigated a realistic model of fluorinated graphene, assuming a random network of the interacting F chains that were observed in the AFM images presented in Fig. 1. The results obtained for the structure shown in Fig. 3b demonstrate that magnetic sites near a single zigzag chain are preserved in disordered networks of densely packed nanoridges. Non-magnetic solution is as much as 582 meV per unit cell less favourable compared to the magnetic configuration in Fig. 3a

The atomic-scale description of the magnetism, as established through *ab initio* calculations, enables us to quantify the experimental results in terms of the spin-coupling strength and the concentration of coupled spins by applying the quantum spin-ladder model in the limit of a strong intra-edge ferromagnetic interaction. The quantum numbers of the ladder eigenstates are the total spin S = 0, 1, …, S_max_ (where S_max _= N_s_ is the largest possible value of the total spin of 2N_s_ spin-½ particles) and its *z*-projection *M*, where ±*M *= 0, 1, …, S. The corresponding eigenenergy (see the Supplementary Material) is as follows


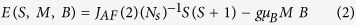


where *J*_*AF*_ is the effective inter-leg coupling constant per spin pair, *g* is the electron gyromagnetic ratio, and *B* is an external magnetic field.

The calculations of the magnetic susceptibility χ are provided in the Supplementary Material, and the resulting temperature dependencies of the susceptibility are depicted in Fig. 3d. As expected for a disordered chain network, none of the single curves derived from Eq. 2 satisfactorily describes the experiment, whereas a combination of two structural descriptions (*N*_*s*_ = 1 and *N*_*s*_ = 3) with the same *J*_*AF*_ value provides a perfect fit to the experimental data (Fig. 4a,b). The *N*_*s*_ = *1* contribution originates from imperfect chains, e.g., the T-junctions of the chains. The presence of a positive χ_0_ term in Eq. 1 indicates that longer chains are also present in the samples, enhancing the paramagnetic background. The magnetic model of the C_2_F stoichiometry is more complicated because of the denser network of fluorine chains on the basal plane, resulting in a 1D-like π-electron system. We speculate that the kink in the temperature curves (Fig. 4c) may be a manifestation of a Peierls-instability-type mechanism, which has been predicted for quasi-one-dimensional even-numbered carbon chains generated in sp^3^-functionalized graphene^28^,^29^. The ferromagnetic-like non-linear component may originate from sublattice imbalance^30^, most likely from odd-numbered π-electron chains^29^. A parallel can be drawn between our results and the persistent reports of room-temperature ferromagnetism in graphene-based samples achieved through the functionalisation^31^,^32^,^33^.

By extracting the strength of the interaction from the χ(T) data and estimating the number of interacting spins, we draw the following quantitative conclusions: (i) the *J*_*AF*_ value for the C_3_F and C_4_F stoichiometries is approximately 450 K (Fig. 4d), and that for C_2_F is approximately 330 K; (ii) the concentration of coupled spins is particularly large, in the range n_s _≈ 10^20^–10^21 ^g^−1^, which corresponds to 1 spin per several CF units (Fig. 4e). This value is 2–3 orders of magnitude greater than previously reported^2^,^34^; besides, the magnetic states induced within the C-sp^2^ nanosegments are strongly correlated and magnetic susceptibility is characteristic of thermal excitation from a nonmagnetic ground state with a spin gap.

Based on various characterisation techniques, thermomagnetic measurements and *ab initio* calculations, a picture emerges in which the quantum confinement of the electronic states, induced by the presence of the fluorine interfaces, enhances the Coulomb interactions and strengthens electron-electron correlations. The 1D nature of these correlated states suppresses long-range order and, in accordance with the theories that account for the quantum nature of edge magnetism, gives rise to magnetic behaviour that is typical of low-dimensional quantum spin systems. The apparent connection between a spin gap and superconductivity has been a source of motivation to search for quasi-one-dimensional ladder materials^35^. Calculations performed for similar but hypothetical structures indicate that, upon either electrical or chemical doping, on/off switching between nonmagnetic and magnetic states might be achieved; such functionality is a prerequisite for spintronics applications^14^. Our results provide the first practical step towards the fabrication of theoretically constructed spin valves^16^ and spin filters^36^ that exploit the differences between the ground and excited states when toggled by the gate bias. Stacked fluorographene can be easily delaminated in organic solvents, which will be a beneficial property for its integration into electronic devices.

## Methods

### Synthesis of C_n_F graphenes

The C_n_F structures were synthesized via the intercalation and fluorination of crystalline graphite. Here, we restrict ourselves to bilayer graphenes prepared from the 2^nd^ stage graphite intercalated compounds. We have similar magnetic results on single-layer graphenes prepared from the 1^st^ stage graphite intercalated compounds. However, due to technological constraints, the single layer graphenes have less perfect structure, leading to 3D graphite-like magnetism which is outside the scope of this paper. During the first stage of synthesis, the graphite platelets were placed in a Teflon flask with a perforated wall; the flask was sealed with a lid and placed in a hermetic reactor containing liquid bromine. After 2 days of exposure of the graphite to Br_2_ vapour at room temperature, an intercalation compound with a composition of C_8_Br was formed. The C_8_Br compound was subsequently transferred to another Teflon flask and placed over a solution of BrF_3_ in Br_2_. The fluorination proceeded at room temperature. The next stage included a subsequent exchange of guest components from the fluorination media to pure Br_2_. The resultant product consisted of fluorinated graphene layers separated by intercalated bromine molecules. During the final stage, Br_2_ was exchanged with other guest molecules. The sample thus obtained was dried under nitrogen flow until a constant mass was achieved, which resulted in uniformly fluorinated graphene bilayers with the C_n_F stoichiometry. Immediately after synthesis, all samples were diamagnetic with a small Curie paramagnetic component. As is known from structural studies, long-term relaxation processes on the scale of approximately one year affect the structures of C_n_F samples. We observed the emergence of both a non-Curie component and an irreversible magnetic response after one to three months of storage under an inert atmosphere. During one year of storage, the non-Curie paramagnetism manifesting spin-ladder magnetism of the samples gradually developed and finally stabilised.

### Characterization methods

A topographic image of the C_3_F surface was obtained using a Nanoscope III atomic force microscope (Digital Instruments, Veeco, Santa Barbara, CA, USA) in a semi-tapping mode under ambient conditions. Magnetic measurements were performed using an MPMS-XL1 superconducting quantum interference device (SQUID)-based magnetic property measurement system manufactured by Quantum Design; the measurements were performed in magnetic fields of −10 kOe to 10 kOe, and the data were collected in the 1.76–300 K range using the ZFC (zero-field cooling), FC (field-cooled cooling) and FCW (field-cooled warming) protocols in the applied fields 10–100 Oe. Data were obtained from samples with a mass of ~0.02 g comprising randomly oriented sub-millimeter flakes (0.4 × 0.3 × 0.02 mm^3^). The samples were maintained under an inert atmosphere to ensure the absence of magnetic effects originating from oxygen.

### Computational Details

All density functional theory (DFT) calculations were performed using the GPAW computer code, based on real space grid implementation of the projector augmented wave (PAW) method. The electronic exchange and correlation effects were described by the Perdew-Burke-Ernzerhof (PBE) functional. An isolated F chain and the network of chains were modelled using unit cells with 128 and 448 carbon atoms, respectively, and a grid spacing of 0.15 Å. Two-dimensional periodic boundary conditions were imposed across the honeycomb sheet. In the direction perpendicular to the surface, we employed open boundary conditions with 9 Å of vacuum separating the graphene layer from the cell boundaries The k-point sampling was restricted to Γ-point. The positions of all atoms were optimized using the BFGS algorithm.

## Additional Information

**How to cite this article**: Makarova, T. L. *et al.* Edge state magnetism in zigzag-interfaced graphene via spin susceptibility measurements. *Sci. Rep.* **5**, 13382; doi: 10.1038/srep13382 (2015).

## Supplementary Material

Supplementary Information

## Figures and Tables

**Figure 1:**
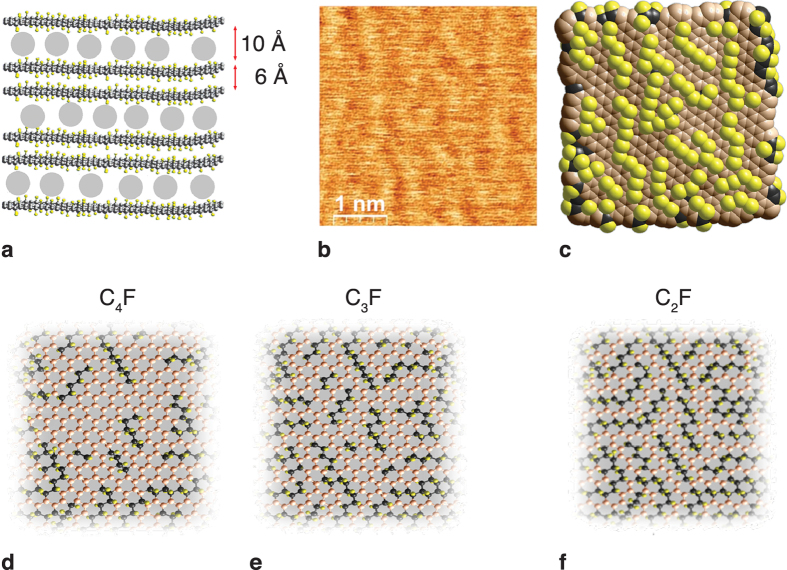
Structure of the C_n_F compounds. (**a**) Side view. The bilayers of fluorinated graphenes are separated by intercalating guest molecules (grey circles). (**b**) Atomic force microscopy (AFM) topographical image of a basal plane of a C_3_F sample. (**c**) Atomistic reconstruction of the AFM image. (**d–f**) Basal-plane structures of samples with continual C-sp^2^ nanosegments in C_4_F (**d**) and in C_3_F (**e**) and isolated C-sp^2^ nanochains (C_2_F) (**f**) plotted according to a set of spectroscopic data^17^,^18^.

**Figure 2:**
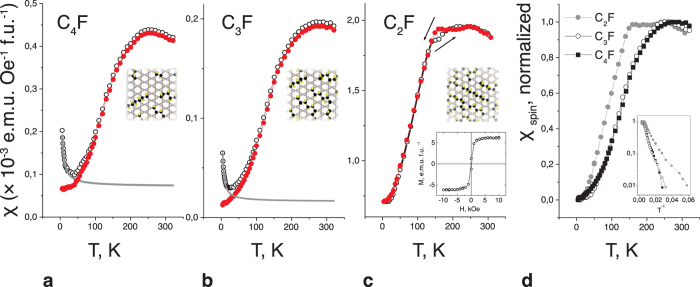
Magnetic susceptibility of the C_n_F samples. (**a,b**) Raw data from the C_4_F (**a**) and C_3_F (**b**) samples measured at an applied magnetic field of 100 Oe upon being heating after zero-field cooling (open circles); the same curves with the Curie contribution (grey) subtracted are represented by filled red circles. (**c**) Raw data from a C_2_F sample measured upon heating (filled circles) and upon cooling (open circles); inset: room-temperature M(H) loop. (**d**) Normalized spin susceptibilities for C_4_F, C_3_F and C_2_F samples; inset: spin susceptibility *vs* reciprocal temperature for these samples.

**Figure 3:**
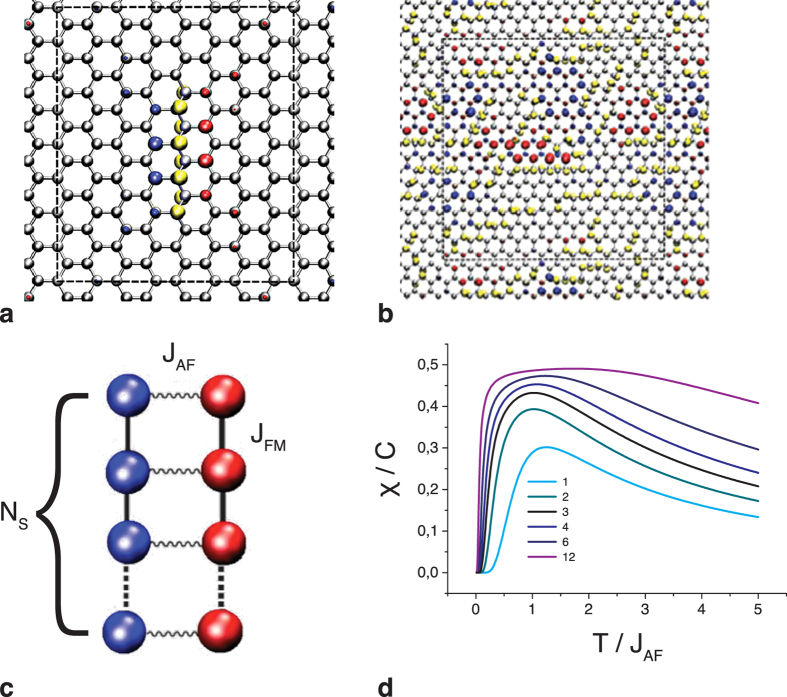
Magnetic units and exchange interactions for the fluorine chains on the graphene basal plane. (**a**) A zigzag chain of fluorine on graphene, with isocontour plots of the spin density obtained from the DFT calculations. Regions with opposite spin orientations are painted in red and blue. C and F atoms are represented by grey and yellow spheres, respectively. (**b**) The calculated spin density for a random network of both zigzag and armchair chains. (**c**) A model of a spin ladder of length N_s_ with an infinitely strong ferromagnetic intra-leg interaction J_FM_ and an antiferromagnetic inter-leg coupling J_AF_. (**d**) A plot of the magnetic susceptibility χ(T,N)/C for the finite size spin ladder *vs* the reduced temperature T/J_AF_. The numbers near the curves correspond to N_s_; the scaling constant is C = (g μB)^2^/3J_AF_, where *g* is the electron gyromagnetic ratio and μ_B_ is the Bohr magneton.

**Figure 4:**
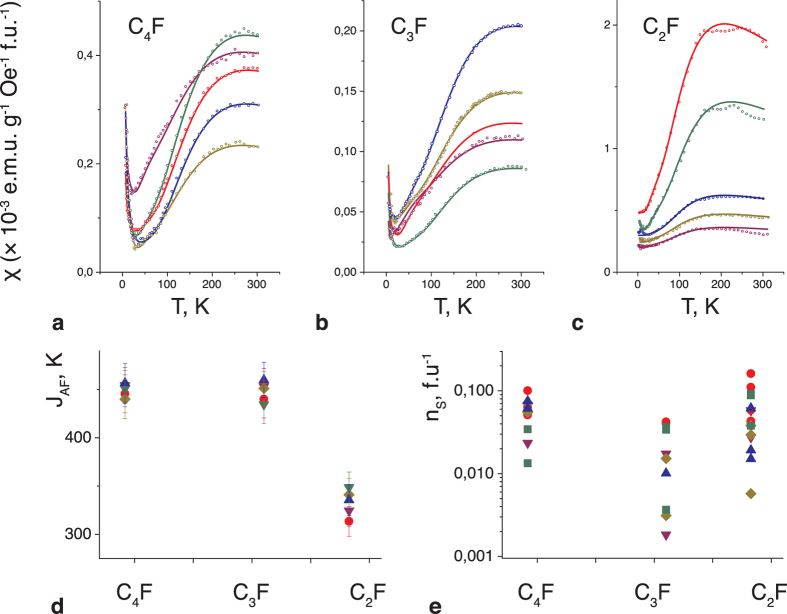
Magnetic susceptibility of the C_n_F structures and the extracted values of the parameters of the spin system. (**a–c)** Temperature dependence of the magnetic susceptibility for samples of three different stoichiometries, as indicated in each panel. The scattered points represent the experimental data, and the lines represent calculations of χ (T, J_AF_, N_S_) using Eq. 2. Different colours correspond to different guest molecules between the graphene planes: red for dichloromethane, blue for dichloroethane, green for acetonitrile, yellow for acetone, and violet for *n*-hexane. (**d**) Exchange-coupling values J_AF_. (**e**) Concentration n_s_ of interacting spins per formula unit *vs* the basal-plane stoichiometry.
