# Supplementary material for: Edge state magnetism in zigzag-interfaced graphene via spin susceptibility measurements
Source: Sci Rep. 2015 Aug 26;5:13382. doi: 10.1038/srep13382 (PMC4549622; doi:10.1038/srep13382)
Supplement: Supplementary Information [file srep13382-s1.pdf]

Supplementary Material for

Edge state magnetism in zigzag-interfaced graphene via spin susceptibility measurements

Supplementary Tables & Supplementary Equations

T. L. Makarova *, A. L. Shelankov, A. A. Zyrianova T. V. Tisnek, A. I. Veinger, A. I. Shames, E. Lähderanta

A. V. Okotrub, L.G. Bulusheva ,G. N. Chekhova, D. V. Pinakov, I. P. Asanov, Ž. Šljivančanin.

*To whom correspondence should be addressed: Tatyana.makarova@lut.fi

This PDF file includes:

Tables S1-S2

Equations S1-S4

Text: 1. Samples

2. Impurity analysis

3. Spin-ladder model

1. Samples. The C_nF samples investigated in the present work are listed in Table S1. Only the samples that were prepared from high-quality, high-purity, crystalline natural graphite with extended defect-free basal planes exhibited the properties described here; in the samples that were prepared from nanographite and pyrolitic graphite, the correlated magnetic effects were masked by a large contribution of paramagnetic spins originating from defects.

Table S1. The stoichiometry and the molar ratio y between the guest molecule and the graphite-fluoride matrix in the investigated samples, all of which had the common formula $(C_2F_xBr_{0.01}\cdot y\text{Guest})_n$, as determined by elemental analysis.

Approximate stoichiometry	Exact stoichiometry	Mole ratio y of the guest molecule				
		CH_3CN	$C_2H_4Cl_2$	CH_2Cl_2	$(CH_3)_2CO$	n-C_6H_{14}
C_2F	$C_2F_{0.92\pm0.01}$	0.136	0.085	0.106	0.097	0.059
C_3F	$C_3F_{1.035\pm0.015}$	0.112	0.076	0.094	0.073	0.048
C_4F	$C_4F_{0.98\pm0.02}$	0.084	0.069	0.071	0.064	0.044

2. Impurity analysis. To exclude the possibility that the results of our investigations could be attributable to the presence of a small amount of reactants or impurities, we performed an impurity analysis using atomic absorption spectrometry (AAS), high-resolution inductively coupled plasma mass spectrometry (HR-ICP-MS), and particle-induced X-ray emission (PIXE). In the as-prepared C_nF samples, both the AAS and HR-ICP-MS analyses indicated the presence of metallic impurities on the order of 10 weight ppm (0.001%) in total. To exclude the effects of sample handling on the magnetic measurements, some samples were subjected to additional analyses immediately following the SQUID measurements. As an example, Table S2 presents the results for the samples that exhibit nonlinear ferromagnetic-like behavior: C_2F-dichloromethane (Ms = 5.5 emu/mol) and C_2F-acetonitrile (Ms= 0.4 emu/mol). The concentrations of magnetic transition metals (Fe, Ni, Co) did not exceed 1 ppm. The diamagnetism of the thermally decomposed fluorocarbons in combination with the results of the elemental chemical analyses precludes any interference from metals in the reported magnetic behavior.

Table S2. The concentrations of metal impurities in the C2F samples that exhibit room-temperature nonlinear ferromagnetic-like behavior. Analysis report № 01TM12-09b (Arcane).

Elements	Concentration in ppm, PIXE		Concentration in ppm, HR ICP MS	
	**M1 (C_2F /dichloromethane)**	**AN1 (C_2F- /acetonitrile)**	**M1 (C_2F /dichloromethane)**	**AN1 (C_2F /acetonitrile)**
Cr	< 0,6	< 0,6	1.10	<0,1
Mn	< 0,5	< 0,5	0.277	<0,1
Fe	< 0,4	1	0.91	1,29
Co	< 0,4	0,45	0.0187	<0,03
Ni	< 0,5	< 0,5	0.471	<0,3
Cu	4,5	5,3	n/a	n/a
Zn	< 0,5	< 0,5	n/a	n/a

3. Spin-ladder model: Magnetic susceptibility of a nanoridge

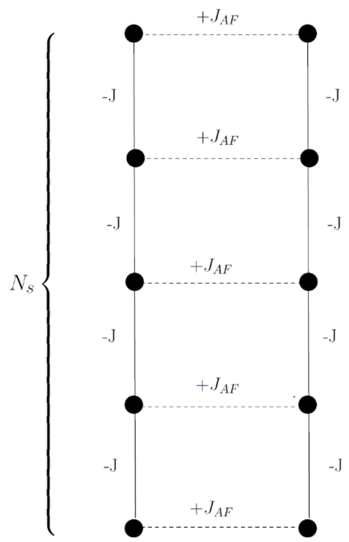

Scheme 1. Spin-ladder model of a nanoridge. The N_s spins on one leg of the ladder correspond to spins localized on "peculiar" states at the graphene edges generated by a fluorine chain. The spins interact ferromagnetically within the legs; J is the coupling strength, and J_{AF} is the strength of the inter-leg antiferromagnetic interaction.

The number of spins is a parameter of the chain. The Hamiltonian of the model reads

$$H = H_l + H_r + H_{lr} \ ,$$

where $H_{l,r}$ is the Heisenberg Hamiltonian of the left or right leg,

$$H_\alpha = -J \sum_{<nm>} \mathbf{S}_{\alpha n} \cdot \mathbf{S}_{\alpha m} \ , \alpha = l, r \ , J > 0 \ ,$$

where $\mathbf{S}_{\alpha n}$ is the spin at site n on leg $\alpha = l, r$ and $<nm>$ represents the nearest neighbours; H_{lr} is the inter-leg interaction:

$$H_{lr} = J_{AF} \sum_n \mathbf{S}_{l,n} \cdot \mathbf{S}_{r,n} \ , J_{AF} > 0. \qquad \text{(S1)}$$

We solve the model in the limit of a strong intra-leg ferromagnetic coupling J and temperatures well below J. The only relevant degree of freedom is the orientation of the collective moments $\mathbf{S}_l = \sum_n \mathbf{S}_{l,n}$ and $\mathbf{S}_r = \sum_n \mathbf{S}_{r,n}$. The system of N_s aligned spin- ½ is in the state with the total spin $\mathbf{S}_{l,r}^2 = s(s+1)$, where $s = \frac{1}{2} N_s$.

The antiferromagnetic interaction of Eq. (S1) in terms of the total spin of the nanoridge is

$$H_{AF} = \frac{1}{2N_s} J_{AF} \mathbf{S}^2 - g\mu_B B S_z \tag{S2}$$

Here, $g = 2$ is the electron g-factor, and μ_B is the Bohr magneton.

The thermodynamic properties of a nanoridge containing N_s spins per side in a magnetic field B can be expressed via the following partition function

$$Z(T,B;N_s) = \sum_{S=0}^{N_s} \sum_{M=-S}^{S} e^{-E(S,M;B,N_s)/k_B T} .$$

In particular, the following result is obtained for the linear magnetic susceptibility χ of the nanoridge:

$$\chi(T;N_s) = \sum_{S=0}^{N_s} P_S(T;N_s)\chi_S(T) , \ \chi_S(T) = \frac{(g\mu_B)^2}{3k_B T} S(S+1) \tag{S3}$$

where $P_S(T;\ N_S)$,

$$P_S(T;N_s) = \frac{1}{Z_0}(2S+1)e^{-\frac{J_{AF}}{2k_B T N_s}S^2} , Z_0 = \sum_{S=0}^{N_s}(2S+1)e^{-\frac{J_{AF}}{2k_B T N_s}S^2} , \tag{S4}$$

represents the probability of the nanoridge being in a state with a total spin S.
